# Immunomodulatory Roles of Polysaccharide Capsules in the Intestine

**DOI:** 10.3389/fimmu.2020.00690

**Published:** 2020-04-15

**Authors:** Samantha A. Hsieh, Paul M. Allen

**Affiliations:** Department of Pathology and Immunology, Washington University School of Medicine, St. Louis, MO, United States

**Keywords:** polysaccharide capsules, adaptive immunity, innate immunity, regulatory T cells, antigen processing, intestinal microbiota

## Abstract

The interplay between the immune system and the microbiota in the human intestine dictates states of health vs. disease. Polysaccharide capsules are critical elements of bacteria that protect bacteria against environmental and host factors, including the host immune system. This review summarizes the mechanisms by which polysaccharide capsules from commensal and pathogenic bacteria in the gut microbiota modulate the innate and adaptive immune systems in the intestine. A deeper understanding of the roles of polysaccharide capsules in microbiota-immune interactions will provide a basis to harness their therapeutic potential to advance human health.

## Introduction

The dynamic interactions between the gut microbiota and immune system determine whether immune tolerance or inflammation and disease develop in the human intestine. One component of bacteria that has been found to play an important role in regulating immune responses in the gut are polysaccharide capsules. Polysaccharide capsules are long polysaccharide chains that form the outermost layer of bacteria and can be several 100 μm thick (1). Capsules are thought to be critical for bacterial fitness and provide protection against many environmental and host factors, especially since they are often the first bacterial structural encountered by the host immune system (2). The study of bacterial capsules had a key role in the early days of molecular biology with the seminal studies of Avery, MacLeod, and McCarty showing that DNA was the genetic material (3). They studied two strains of *Streptococcus pneumoniae*, smooth (S) and rough (R), which were virulent and avirulent, respectively. The S strain produced a capsule, whereas the R strain did not. They were able to transform the R strain into an avirulent S strain using DNA from the S strain, thereby proving that DNA was the genetic material (3).

One striking feature of the capsules in general is their chemical and structural diversity within a given bacterial species. They can be composed of different monosaccharides, which can also vary in their stereoisomers (D or L), number of carbon molecules forming the sugar ring structure (furanose or pyranose), and configuration of the anomeric center of each sugar (Cα or Cβ) (4). These polysaccharides can be further diversified by branching and glycosidic linkage patterns (α or β), covalent coupling to other biological molecules such as proteins and lipids, and forming secondary structures (5). This astronomical diversity likely equips polysaccharide capsules with many different functions that enhance bacterial fitness.

Polysaccharide capsules have been shown to enhance bacterial survival in the gut by multiple mechanisms. Capsule composition varies with diet and different capsules may provide optimal access to various nutrients and more efficient use of the bacterial cell's resources (5). Another role of polysaccharide capsules in the gut microbiota is exclusion of pathogens as capsule expression has been shown to alter disease symptoms as well as bacterial attachment to host cells and thus bacterial abundance (5). Capsules can be degraded by other microbes, which can be either beneficial or detrimental to the bacteria. In mutualistic relationships, they can be used as a nutrient source for neighboring bacteria. However, capsules can also be degraded by competing bacteria, reducing bacterial fitness. Capsules have also been shown to provide protection against bacteriophages (5). In addition, polysaccharide capsules can alter immune responses to bacteria. Interestingly, even minor differences between capsules can completely alter the immune response as each is recognized as immunologically distinct (4).

Some bacteria, especially the *Bacteroides* species, express polysaccharide capsules that are phase variant and are able to switch between different capsules (6). This phase variance arises because each polysaccharide can regulate its expression using a reversible on-off phenotype, which is controlled by inversions of the DNA promoter regions that lie upstream of their polysaccharide biosynthesis loci (7). These inversions place the promoters in either the correct or incorrect orientation for transcription of the downstream polysaccharide biosynthesis genes. Phase variation is thought to equip bacteria to adapt and survive in different niches in the gut and may be especially advantageous in the face of immune pressure by enabling bacteria to alter their surface antigenicity (5).

The two main types of polysaccharide capsules are exopolysaccharides (EPSs) and capsular polysaccharides (CPSs). EPSs contain glycans that are loosely associated with microbial cell surfaces, while CPSs are composed of glycans that are firmly attached to the cell surface (5). However, determining whether or not glycans are attached to the cell surface is difficult, so these terms are often used interchangeably. In this review, we will use the term microbially produced glycans (MPGs) to encompass both CPSs and EPSs (5). MPGs have been found in a variety of Gram-negative and Gram-positive bacteria including *Escherichia coli, Neisseria meningitides, Haemophilus influenza, Staphylococcus aureus*, and *Streptococcus pneumoniae* and are critical for serological classification and vaccine development (2). Although the roles of MPGs in the gut microbiota are less understood, there have been many recent advances in understanding the interplay between bacterial MPGs and the host immune system in the intestine. Here we review the current literature on the immunomodulatory roles of MPGs in the intestine on both innate and adaptive immune responses. A summary of the effects of different MPSs, their capsule composition, immune cell target, and mechanisms of action are detailed in Table 1.

**Table 1:** Summary of the effects of bacterial polysaccharide capsules in the intestine on the immune system.

**Bacteria**	**Gram stain**	**Commensal/Pathogen**	**MPG**	**Capsule composition**	**Immune cell target**	**Mechanism**	**Targeted immune pathways**	**References**
*Bacillus subtilis*	Gram +	Commensal	EPS	Mannose (88%), glucose (11.9%), *N*-acetylglucosamine (0.1%); Primary linkages: 2,6-mannose (31.8%), terminal mannose (29.9%), 3-mannose (15%), 2-mannose (4.7%), 6-mannose (4.7%), 6-glucose (3.7%), and terminal glucose (3.5%)	Macrophages	Signals through TLR4/MyD88 pathway to induce M2 macrophages, which inhibit CD4+ T cells via TGF-β and CD8+ T cells via TGF-β and PD-L1; protects against C. rodentium-induced colitis	Macrophage skewing; T cell activation	(8, 9)
*Bacteroides cellulosilyticus*	Gram −	Commensal	CPS	Acetamido-amino-2,4,6-trideoxygalactose (AATGal) amino sugar; zwitterionic	Monocytes	Induces IL-10 and CD25+FoxP3+CD127-CTLA-4+ Tregs; attenuates TNBS-induced colitis	Cytokine production; T cell activation	(10)
*Bacteroides fragilis*	Gram −	Commensal	CPS: PSA	Tetrasaccharide repeating unit containing 4,6-pyruvate attached to a d-galactopyranose, 2,4-dideoxy-4-amino-d-FucNAc, d-N-acetylgalactosamine, and d-galactofuranose with one positively charged free amine and one negatively charged carboxylate; zwitterionic	Dendritic cells	Enhances antigen presentation by upregulating MHC II, CD80, and CD86; phagocytosed by APCs and displayed on MHC II to activate CD4+ T cells in a TLR2-dependent manner; corrects CD4+ T cell deficiencies and T_H_1/T_H_2 imbalance in germ-free mice by upregulating the production of IFN-γ+ T_H_1 T cells through CD11c+ DCs and the IL-12/STAT4 pathway; represses T_H_17 responses; induces IL-10 producing FoxP3+ Tregs in a TLR2-dependent manner; protects against TNBS-induced colitis and *Helicobacter hepaticus*-induced colitis; OMVs	Antigen presentation; T cell activation	(11–23)
*Bacteroides thetaiotaomicron*	Gram −	Commensal	CPS: CPS 1-8	CPS1: 22% N-acetyl-glucosamine, 33% glucose, 9% mannose, 36% galacturonic acid CPS2: 8% N-acetyl-glucosamine, 85% glucose, 7% mannose!!!break!!! CPS3: 18% N-acetyl-glucosamine, 42% glucose, 34% mannose, 6% galacturonic acid!!!break!!! CPS4: 61% N-acetyl-glucosamine, 21% glucose, 18% galacturonic acid!!!break!!! CPS5: 10% N-acetyl-galactosamine, 7% N-acetyl-glucosamine, 27% galactose, 49% glucose, 8% mannose!!!break!!! CPS6: 23% N-acetyl-galactosamine, 8% N-acetyl-glucosamine, 37% galactose, 23% glucose, 6% mannose, 3% glucuronic acid!!!break!!! CPS8: 19% N-acetyl-glucosamine, 2% galactose, 63% glucose, 14% mannose, 1% glucuronic acid	Dendritic cells, macrophages	CPS2, CPS4, CPS5, CPS6, and WT are more resistant to complement; CPS1-6 inhibit APC phagocytosis and antigen presentation likely due to increased capsule thickness and decrease IL-6 and TNF-α production in a MyD88-dependent manner; CPS5 promotes evasion of IgA responses; CPS1 represses polyclonal and antigen-specific T cell activation and differentiation to IFN-γ+ IL-17A+ T cells; OMVs	Complement evasion; phagocytosis; antigen presentation; cytokine production; evasion of antibody responses; T cell activation	(24, 25)
*Bifidobacterium bifidum*	Gram +	Commensal	CPS	Mixture of four neutrally charged cell surface β-glucan/galactan (CSGG) polysaccharides: β-(1 → 6)-glucan, β-(1 → 4)-galactan, β-(1 → 6)-galactan, β-galactofuranan and starch	Dendritic cells	induces IL-10 and FoxP3+ Tregs through TLR2-mediated mechanism on DCs; attenuates colitis in T cell transfer model of colitis	Cytokine production; T cell activation	(26, 27)
*Bifidobacterium breve*	Gram +	Commensal	EPS	Glucose, galactose and/or the N-acetylated versions of these two sugars	B cells	Decreases numbers of B cells and antigen-specific total Ig, IgG3, IgG1, IgG2a, and fecal IgA titers; elicits weaker antibody responses by masking surface antigens	B cell activation; masks surface antigens	(28, 29)
*Bifidobacterium longum*	Gram +	Commensal	EPS	Branched hexasaccharide repeating unit with two galactoses, two glucoses, galacturonic acid, and the unusual sugar 6-deoxytalose	Neutrophils, macrophages, dendritic cells, NK cells	Decrease IFN-γ, IL-12, TNF-α, IL-17, IL-6 production; prevents phagocytosis; represses T_H_17 recruitment; protects against T cell transfer model of colitis	Cytokine production; phagocytosis; T cell activation	(30–32)
*Campylobacter jejuni*	Gram −	Pathogen	CPS	Heptoses in unusual configurations (e.g., ido, gulo, and altro) and non-stoichiometric modifications on the sugars, including ethanolamine, aminoglycerol, and O-methyl phosphoramidate (MeOPN)	Dendritic cells, macrophages	Blocks antibody binding and activation of complement; decreases activation of TLR4 and production of IL-1β, IFN-γ, and IL-6	Complement evasion	(33, 34)
Enteropathogenic *Escherichia coli*	Gram −	Pathogen	CPS: Gp 4 capsule	Linear tetrasaccharide made of L-fucose, D-galactose and two N-acetyl-galactosamines	Human α-defensin 5	Uses its anionic charges to prevent cationic human α-defensin 5 from reaching the bacterial membrane	Innate immune evasion of antimicrobial peptides	(35)
*Faecalibacterium prausnitzii*	Gram +	Commensal	EPS	Unknown	Dendritic cells	Decreases IL-12p70 and IFN-γ and increases IL-10 secretion through TLR2 signaling by *Lactobacillus plantarum*; induces Foxp3+ CD4+ T cells	Cytokine production; attenuates DSS-colitis	(36)
*Helicobacter hepaticus*	Gram −	Pathobiont	CPS	α-mannose and α-glucose sugars	Macrophages	Signals through TLR2/MyD88 pathway to activate MSK/CREB pathway and induce IL-10	Innate immune tolerance	(37)
*Lactobacillus fermentum*	Gram +	Commensal	EPS	MMMP1: main chain of 1,6-β-D-Galfs with non-stoichiometric 2-O-glucosylation. MMMP2: a repeat unit → 3)-β-D-Glcp-(1 → 3)-β-D-Galf-(1 → 6)-[2-α-D-Glcp]β-D-Galf-(1 →	Unknown	Induces IgA production	Enhances IgA production	(38)
*Lactobacillus johnsonii*	Gram +	Commensal	EPS	EPS-1: branched dextran with every backbone residue substituted with a 2-linked glucose unit and polysaccharides partially occupied by 1-phosphoglycerol and O-acetyl groups; EPS-2: repeating unit with−6)-α-Glcp-(1–3)-β-Glcp-(1–5)-β-Galf-(1–6)-α-Glcp-(1–4)-β-Galp-(1–4)-β-Glcp-(1- and polysaccharides partially occupied by single O-acetyl group	Unknown	Masks cell surface epitopes from antibodies	Masks surface antigens	(39)
*Lactobacillus kefiranofacien*	Gram +	Commensal	EPS	Equal proportions of glucose and galactose		Induces IgA production	Enhances IgA production	(40)
*Lactobacillus rhamnosus* GG	Gram +	Commensal	EPS	70% galactose, 19% rhamnose, and 10% glucose	LL-37/human cationic protein 18; complement	Resists cationic LL-37/human cationic protein 18 by forming protective shield with long and neutral EPS; protects against complement activation and lysis via lack of mannose	Innate immune evasion of antimicrobial peptides; complement evasion	(41, 42)
*Leuconostoc mesenteroides*	Gram +	Commensal	EPS	Glucose and fructose	Unknown	Induces IgA production, retinoic acid synthase, and TGF-β; increases number of CD4+ and CD8+ T cells	Enhances IgA production; cytokine production; T cell activation	(43)
*Pediococcus parvulus*	Gram +	Commensal	EPS	2-substituted (1,3)-β-d-glucan	Macrophages	Decreases TNF-α and IL-8 production	Cytokine production	(44)
*Salmonella enterica* serovar Typhimurium	Gram −	Pathogen	CPS: Gp 4 cp (O-ag CPS)	Repeating units of glucose, mannose, and galactose	Complement; macrophages	Decreases C3 surface deposition; decreases production of IL-6 and TNF-α in a TLR-dependent manner	Complement evasion; cytokine production	(45–47)
*Salmonella* Typhi	Gram −	Pathogen	CPS: Vi	Homopolymer of (1,4)-2-acetamido-3-*O*-acetyl-2-deoxy-α-D-galacturonic acid	T cells	Represses T cell responses by binding to T cells through the prohibitin complex and inhibiting IL-2 secretion; prevents C3 deposition, phagocytosis, and complement receptor 3-mediated clearance	Represses T cells; complement evasion	(48, 49)
*Shigella sonnei*	Gram −	Pathogen	CPS: Gp 4 cp (O-ag CPS)	High molecular weight polysaccharide containing FucNAc4N and L-AltNAcA residues in 1:1 ratio	Complement	Resists direct complement-mediated killing	Complement evasion	(50)
*Streptococcus thermophiles*	Gram +	Commensal	EPS	12.9% rhamnose, 26% glucose, 60.9% galactose, 0.25% mannose	Unknown	Decreases IFN-γ, IL-6, and TNF-α production	Cytokine production	(51)
*Vibrio cholerae*	Gram −	Pathogen	CPS (O-antigen CPS)	Polymerized O-antigen subunits composed of N-acetylglucosamine, N-acetyl-quinovosamine, galacturonic acid, galactose andcolitose	Complement	Resists complement-mediated bacteriolysis likely by promoting binding of negative regulatory proteins and inhibiting efficient complement fixation at the bacterial surface	Complement evasion	(52, 53)

*Microbially produced glycan (MPG), exopolysaccharide (EPS), capsular polysaccharide (CPS), antigen presenting cell (APC), medium molecular mass exopolysaccharide (MMMP1), and 2,4,5-trinitrobenzene sulfonic acid (TNBS)*.

## MPGS Modulate Innate Immune Responses in the Intestine

The innate immune system is the first line of defense against many pathogens, and MPGs have been shown to be critical for regulating innate immune responses in the gut. One mechanism the innate system uses to combat bacterial infection is the production of antimicrobial peptides such as defensins and cathelicidins by the intestinal epithelium. MPGS can block the bactericidal activity of antimicrobial factors, enabling bacteria to evade these innate immune responses. For example, wild-type (WT) enteropathogenic *Escherichia coli* (EPEC) was more resistant to human α-defensin 5 (HD-5) than an unencapsulated EPEC mutant (35). An unencapsulated EPEC strain could be protected from HD-5 killing by the addition of exogenous EPEC polysaccharide, suggesting that the EPEC capsule likely binds HD-5 and traps it before it reaches the bacterial membrane (35). Similarly, EPS from the probiotic *Lactobacillus rhamnosus* GG (LGG) protected LGG from the LL-37/human cationic protein 18 from the cathelicidin family (42). Viability of an EPS^−^ LGG strain was reduced in the presence of LL-37, while an EPS^+^ LGG strain had no change in viability. In addition, growing LGG in the presence of subinhibitory concentrations of LL-37 induced EPS expression, further demonstrating that EPSs protect microbes from antimicrobial peptides (42).

Another method the innate immune system uses to recognize and destroy bacteria is complement. MPGs can block the deposition of complement on bacterial surfaces, which prevents bacteria from being targeted for destruction by the immune system. EPS from LGG also protected against complement-mediated lysis as an EPS^−^ LGG strain, but not an EPS^+^ LGG strain, had a reduction in viability after incubation with normal human serum (42). Although WT *Salmonella enterica* serovar Typhimurium was resistant to killing by normal human serum, an EPS^−^ strain had increased serum sensitivity as well as faster C3 surface deposition (46). Similarly, an unencapsulated strain of *Shigella sonnei* was more vulnerable to complement than WT and also had a reduced ability to disseminate peripherally (50). *Vibrio cholera's* CPS also protects against complement-mediated bacteriolysis and strains isolated from patients that contained less capsular material were more susceptible to the bactericidal activity of serum (53, 54). The CPS of *Campylobacter jejuni* blocks antibody binding and activation of complement and non-stoichiometric *O*-methyl phosphoramidate (MeOPN) modifications at the 4 position of galactose has been shown to be the most critical to complement resistance (33). An acapsular strain of the Gram-negative symbiont *Bacteroides thetaiotaomicron*, which lacks expression of all *B. thetaiotaomicron* CPSs, also had a decrease in survival after treatment with normal human serum compared to WT, further suggesting that CPSs can enable bacteria to circumvent complement (25). Interestingly, *B. thetaiotaomicron* strains that singly expressed different CPSs had varying susceptibilities to normal human serum as CPS2, CPS4, CPS5, CPS6, and WT were more resistant to complement than CPS1, CPS3, CPS7, and CPS8 (25). These findings demonstrate that MPGs can protect bacteria by inhibiting complement deposition on the surface of bacteria, which prevents complement-mediated lysis as well as uptake by phagocytic cells.

MPGs can also directly block bacterial uptake by innate immune cells, which prevents phagocytic cells from killing bacteria and antigen presenting cells from presenting antigenic peptides on the surfaces that may activate the adaptive immune system. For example, *B. thetaiotaomicron* with anti-stimulatory CPSs such as CPS1 are poorly uptaken by bone-marrow macrophages (BMDM) and splenic CD11c+ dendritic cells (DCs) (24). In contrast, *B. thetaiotaomicron* expressing pro-stimulatory CPSs such as CPS8 or Acap are readily phagocytosed. These differences in phagocytosis may be partly explained by the thicknesses of the *B. thetaiotaomicron* CPSs as anti-stimulatory CPSs tended to be thicker than pro-stimulatory CPSs. In support of these findings, *B. thetaiotaomicron* expressing anti-stimulatory CPSs were cleared less effectively from the peritoneal cavity than *B. thetaiotaomicron* expressing pro-stimulatory CPSs *in vivo*, likely because anti-stimulatory CPSs inhibited uptake by peritoneal innate immune cells (24). In addition, innate immune phagocytes cultured with *B. thetaiotaomicron* expressing anti-stimulatory CPSs were poor antigen presenters to the adaptive immune system. *Bifidobacterium longum*'s EPS also inhibited macrophage phagocytosis (32). In contrast, other MPGs can promote the maturation of innate immune cells so that they serve as better antigen presenting cells (APCs) to the adaptive immune system. For example, the polysaccharide A capsule (PSA) expressed on *Bacteroides fragilis* upregulates MHC II expression on CD11c+ dendritic cells as well as the co-stimulatory molecules CD80 and CD86, which enhances antigen presentation (18).

Innate immune responses to bacteria can also be thwarted by MPGs that promote immune tolerance by inducing innate immune cells to release anti-inflammatory cytokines in the intestine. A large soluble polysaccharide released by the Gram-negative *Helicobacter hepaticus* induced an anti-inflammatory M2 gene signature as well as IL-10 production in intestinal macrophages through TLR2, mitogen and stress-activated protein kinase (MSK), and cyclic AMP response-element binding protein (CREB) (37). Similarly, the EPS from the Gram-positive probiotic *Bacillus subtilis* elicited anti-inflammatory M2 macrophages through TLR4, which was required for EPS-mediated protection from *Citrobacter rodentium*-induced colitis (8, 9) and limited inflammation during *Staphylococcus aureus* infection (55).

Other MPGs protect bacteria from innate immune responses by reducing the production of pro-inflammatory cytokines by innate immune cells. Anti-stimulatory CPSs expressed on *B. thetaiotaomicron* decreased the levels of the pro-inflammatory cytokines IL-6 and TNF-α produced by macrophages and dendritic cells (DCs) in the presence of *B. thetaiotaomicron* (24). Neutrophils, macrophages, and NK cells each produced less IFN-γ, IL-12, and TNF-α in mice treated with EPS^+^ vs. EPS^−^ *Bifidobacterium breve* (*B. breve*) (28). EPS from *Bifidobacterium longum* also induced lower levels of IL-12p70, IFN-γ, and IL-17 in human peripheral blood mononuclear cells and IL-17, IL-6, and TNF-α in human monocyte-derived dendritic cells compared to an EPS^−^*B. longum* strain (31). In support of these findings, adding isolated EPS to PBMC cultures with EPS^−^ *B. longum* reduced the secretion of IL-12p70 and IFN-γ (31). Neutrally charged EPS from the putative probiotic *Streptococcus thermophiles* also decreased the production of the pro-inflammatory cytokines IFN-γ, IL-6, and TNF-α in DSS-induced colitis (51). The production of TNF-α and IL-8 production by human monocyte-derived macrophages was reduced in the presence of an EPS^+^ *Pediococcus parvulus* strain compared to an EPS^−^ strain (44). *Campylobacter jejuni*'s CPS decreases activation of TLR4 and production of pro-inflammatory IL-1β, IFN-γ, and IL-6 (33). Expression of *Salmonella*'s Vi capsular polysaccharide led to a decline in IL-6 and TNF-α secretion by BMDM in a TLR-dependent manner (47). In addition, an extracellular polymeric matrix from *Faecalibacterium prausnitzii* reduced the secretion of pro-inflammatory IL-12p70 in a TLR2-dependent manner in human monocyte-derived DCs cultured with *Lactobacillus plantarum* compared to *L. plantarum* alone (36). These results demonstrate that the gut microbiota also uses MPGs to inhibit innate immune responses by decreasing the production of pro-inflammatory cytokines and increasing the production of anti-inflammatory cytokines by innate immune cells. This induction of innate immune tolerance by MPGs may represent one way innate immune cells are able to distinguish between pathogens and commensal bacteria, although this mechanism can also be co-opted by pathogens to evade the innate immune system.

## MPGS Modulate Adaptive Immune Responses in the Intestine

The host immune system also consists of adaptive immunity, especially B and T cells, which protect the intestine from bacterial pathogens in an antigen-specific manner and induce immunological memory that leads to enhanced immune responses to consequent encounters with pathogens. Bacteria also encounter the adaptive immune system in the intestine, and the ability of MPGs to evade adaptive immune responses is crucial to their fitness and survival in the gut. One mechanism MPGs use to modulate the adaptive immune system is regulating the interactions between B cells, antibodies, and bacteria. Many MPGs inhibit B cell and antibody responses to bacteria. For example, EPS^+^ *B. breve*-treated mice had decreased numbers of B cells as well as lower antigen-specific total Ig, IgG3, IgG1, IgG2a, and fecal IgA titers (28). EPS^+^ *B. breve* persisted longer than EPS^−^ *B. breve* in WT mice, but this difference was abolished in B cell-deficient mice, suggesting that EPS on *B. breve* modulates B cells and plays a role in evading adaptive immune responses. In addition, EPS^+^ *B. breve* weakly agglutinated with anti-EPS serum, while EPS^−^*B. breve* strongly agglutinated with serum from EPS^−^-treated mice but not anti-EPS serum, demonstrating that MPGs can mask surface antigens from detection and thereby elicit weaker antibody responses (28). EPS from the potential probiotic *Lactobacillus johnsonii* also protected cell surface epitopes from exposure to antibodies as an EPS^−^ strain of the potential probiotic *Lactobacillus johnsonii* bound to more polyclonal antibody raised against WT *L. johnsonii* than an EPS^+^ strain. A mutant that overexpressed *L. johnsonii* EPS had similar levels of bound antibody compared to wild type, indicating that the *L. johnsonii* EPS itself is poorly recognized by the immune system (39). In germ-free mice that were colonized with all 8 single CPS-expressing *B. thetaiotaomicron* strains, higher levels of IgA production correlated with increased abundance of the CPS5-expressing strain, suggesting that some CPSs such as CPS5 may promote evasion of IgA responses (25). In contrast, other MPGs promote the production of antibodies as EPSs from the probiotic lactic acid bacteria *Lactobacillus kefiranofaciens, Leuconostoc mesenteroides*, and *Lactobacillus fermentum* induced IgA production in the intestine, which may enhance the protective nature of intestinal barrier (38, 40, 43). Thus, depending on the MPG, MPGs can either inhibit or bolster B cell and antibody responses in the intestine.

Many MPGs can also modulate T cell responses in the intestine, including by functioning as T cell antigens and by regulating both polyclonal and antigen-specific T cells. Most polysaccharides are classically considered to be T cell-independent antigens that do not induce the activation of helper T cells that stimulate Ig class switching in B cells or immunologic memory (56). Instead, polysaccharides must be coupled to protein carriers in order to enlist T cell help and induce IgG antibodies and memory B cells such as in vaccines (6). Although carbohydrates are not usually T cell antigens, zwitterionic polysaccharides (ZPSs), which contain both a positive and negative charge, are unique MPGs that have been shown to regulate T cells by directly acting as a T cell antigen. The best studied zwitterionic MPG is the *B. fragilis* PSA. Although PSA is a carbohydrate, PSA can be presented by APCs to activate T cells (11, 12). PSA is taken-up into APC endosomes, processed by inducing nitric oxide production through TLR2, and is displayed on major histocompatibility complex (MHC) II to activate CD4+ T cells in mice (14, 16, 17, 23). In human dendritic cells (DCs), the C-type lectin dendritic cell-specific intercellular adhesion molecule-3-grabbing non-integrin (DC-SIGN) is the main receptor for PSA (57). PSA's ability to directly stimulate TLR2 has recently come under debate as PSA is not a typical TLR2-stimulating molecular structure and one study suggests that the lipoproteins in *B. fragilis* glycoconjugate fractions are responsible for stimulating TLR2, not PSA (58). PSA is currently the only CPS that has been shown to function as an antigen and is directly presented to T cells.

MPGs have also been shown to be critical for maturation of the host immune system, especially the development of T cells. Although germ-free mice that lack the bacterial microflora are known to exhibit immunological defects, *B. fragilis* PSA was sufficient to correct these T cell deficiencies (18). Mono-colonizing germ-free mice with WT *B. fragilis* or purified PSA, but not PSA-deficient *B. fragilis*, corrected the CD4+ T cell deficiencies in germ-free mice. Although PSA had no effect on the proportions of CD8+ T cells or CD19+ B cells, PSA also corrected the T_H_1/T_H_2 imbalance in germ-free mice by upregulating the production of IFN-γ+ T_H_1 T cells through CD11c+ DCs and the IL-12/STAT4 pathway (18).

MPGs can also modulate the activation of polyclonal T cell responses, especially by directing T cell differentiation. Most T cell effects of MPGs that have been studied induce a state of immune tolerance by suppressing T cell responses. In the Powrie model of colitis, EPS^+^ *B. longum* induced fewer IL-17A+ lymphocytes and protected against colitis compared to EPS^−^ *B. longum* (31). *Salmonella* Typhi capsular polysaccharide Vi repressed T cell responses by binding to T cells through the prohibitin complex and inhibiting IL-2 secretion (48). *B. thetaiotaomicron* expressing anti-stimulatory CPSs were also found to activate polyclonal T cells in germ-free mice more weakly than *B. thetaiotaomicron* expressing pro-stimulatory CPSs (24). In addition, *B. fragilis* PSA repressed T_H_17 cell responses, which is required for *B. fragilis* colonization, as *B. fragilis* lacking PSA was unable to restrain T_H_17 cell responses through TLR2 in contrast to WT *B. fragilis* (21). Many MPGs also direct polyclonal T cells to differentiate into regulatory T cells (T_regs_) that often suppress effector T cells as well as intestinal inflammation. *B. fragilis* PSA induces the differentiation of IL-10 producing FoxP3+ T_regs_ in mice in a TLR2-dependent manner, which can protect against TNBS-induced colitis and experimental colitis induced by *Helicobacter hepaticus* (15, 19, 20, 22). PSA can also generate human IL-10 producing T_regs_ *in vitro* (22, 59). Additionally, zwitterionic MPGs on other bacteria can stimulate more IL-10 and higher proportions of CD4+ FoxP3+ regulatory T cells than non-ZPS strains (10). Non-ZPSs can also promote the production of T_regs_ as neutrally charged cell surface β-glucan/galactan (CSGG) polysaccharides from *Bifidobacterium bifidum* induced T_reg_ cells in the intestine and suppressed inflammation in a T cell transfer model of colitis (27). Interestingly, MPGs on outer membrane vesicles (OMVs), which are released by many Gram-negative bacteria, can also regulate T cell responses similarly to MPGs on whole bacteria. *B. thetaiotaomicron* OMVs that singly expressed anti-stimulatory CPSs poorly stimulated T cells compared to *B. thetaiotaomicron* OMVs that singly expressed pro-stimulatory CPSs just like CPSs on whole *B. thetaiotaomicron* (24). *B. fragilis* OMVs that contained PSA induced more IL-10 production and CD4+ FoxP3+ Tregs compared to OMVs that lacked PSA, which also corresponded with the PSA effects observed on whole *B. fragilis* (60).

In addition to modulating polyclonal T cell responses, MPGs can regulate T cell responses to dominant antigens. Using a *B. thetaiotaomicron*-specific CD4+ T cell called BθOM, *B. thetaiotaomicron* expressing anti-stimulatory CPSs was found to weakly activated BθOM T cells while *B. thetaiotaomicron* expressing pro-stimulatory CPSs strongly activate BθOM T cells *in vitro* and *in vivo* (24). CPSs on *B. thetaiotaomicron* also directed the differentiation of antigen-specific T cells as BθOM T cells in the colon differentiated into more IFN-γ+ IL-17A+ T cells in the presence of *B. thetaiotaomicron* containing pro-stimulatory CPSs than anti-stimulatory CPSs (24). These findings demonstrate that by altering MPGs, bacteria can also modulate adaptive immune responses to their dominant antigens.

## Discussion

MPGs play critical roles in regulating the immune responses to the microbiota in the intestine. Many MPGs enable bacteria to evade innate and adaptive immune responses by forming protecting shields around bacteria. For example, MPGs can protect bacteria from antimicrobial factors, complement deposition, and phagocytosis by innate immune cells. MPGs also promote immune tolerance by inducing the innate immune system to produce more anti-inflammatory cytokines or fewer pro-inflammatory cytokines as well as maturation of innate immune cells to make them better APCs for the adaptive immune system. Adaptive immune responses can also be modulated by MPGs as MPGs can block B cell and antibody responses, especially by masking surface antigens. In addition, MPGs, even on OMVs, can regulate T cell responses by directly serving as the T cell antigen or by controlling the activation and differentiation of polyclonal and antigen-specific T cells.

MPGs are critical components of the gut microbiota, and deciphering the roles of MPGs in microbiota-immune interactions in the intestine is crucial for improving human health. Despite the progress in understanding how MPGs modulate immune responses to intestinal bacteria, many immunoregulatory functions of MPGs are still poorly understood. For example, why some non-zwitterionic MPGs are anti-stimulatory whereas others are pro-stimulatory is not known. In addition, bacteriophages can regulate the immune system (61) and MPG-specific bacteriophages have been identified (62), but the interactions between MPG-specific bacteriophages, MPGs, and the immune system remain to be elucidated. Progress in this area has been hampered by the complex chemical structures of MPGs and additional analyses are required to decipher the MPG structures and the mechanisms by which they regulate the immune system. Given the astronomical diversity of MPGs, MPGS likely have many more roles in modulating immune responses that have yet to be discovered. More studies are needed expand the known lexicon of bacterial polysaccharide-immune system interactions, and they may even lead to the discovery of new, bioactive CPS for potential use as therapeutics to improve human health. (13, 26, 29, 30, 32, 34, 41, 45, 49, 53).

## Author Contributions

SH and PA conceived the topic of this review. SH wrote the review and PA edited it.

### Conflict of Interest

The authors declare that the research was conducted in the absence of any commercial or financial relationships that could be construed as a potential conflict of interest.
